# Sublingual Dermoid Cyst: A Diagnostic Challenge During Clinical Examination

**DOI:** 10.1155/crot/8133416

**Published:** 2025-06-12

**Authors:** Ali Hussein Habeeb, Ayad Ahmad Mohammed

**Affiliations:** ^1^Department of Surgery, Azadi Teaching Hospital, Duhok Directorate General of Health, Duhok, Iraq; ^2^Department of Surgery, College of Medicine, University of Duhok, Duhok, Kurdistan Region, Iraq

**Keywords:** cholesterol crystals, dermoid cyst, epidermoid cyst, teratoid cyst

## Abstract

**Introduction:** Epidermoid cysts are benign lesions that may occur in many different sites of the body. They are classified into 3 types: epidermoid cysts when the lining presents only epithelium, dermoid cysts when skin adnexa are found, and teratoid cysts when mesodermal elements are present.

**Case Presentation:** A 13-year-old boy presented with a gradually enlarging sublingual mass over a period of 4 years. The mass was painless at start but in the last 3 months, it was associated with pain and difficulties during eating and dysarthria. The general examination was unremarkable and examination of the oral cavity showed a 5∗6 cm mass in the right side of the tongue and within the tongue. The mass was soft and nontender, fluctuation was positive, there was no pulsation over the mass, transillumination was negative, and other parts of the oral cavity were normal. Neck examination was also normal with no enlargement of the cervical lymph nodes. Aspiration of the lesion showed turbid yellow fluid (keratin like substance) with no blood. Complete surgical excision of the cyst was done, which was dermoid cyst of the tongue containing hair and fat. The patient was discharged on the same day of surgery with no postoperative events.

**Conclusion:** Dermoid cyst of the tongue is an extremely rare condition. High index of suspicion is required for the diagnosis. Aspiration of the cyst helps in the diagnosis due to the typical yellow color because of cholesterol contents. The prognosis is excellent after complete surgical excision.

## 1. Introduction

Epidermoid cysts are benign lesions that may occur in many different sites of the body. The head and neck may be affected in less than 7% of the cases and the oral cavity is affected in less than 1% of the cases. The cysts can be classified into 3 main types depending on the lining and the contents into epidermoid cysts when the lining presents only epithelium partly keratinized epithelium, dermoid cysts when skin adnexa are found within the cyst like hair follicles, hair, sweat, and sebaceous glands, and teratoid cysts containing mesodermal elements such as bone, muscle, respiratory and gastrointestinal tissues, and fibrous capsules in addition to skin appendages [1, 2].

The term dermoid cyst is generally applied to all these 3 types although they have different histopathological types. Dermoid cysts most commonly occur in the testes and ovaries, and the most common site in the head and neck is the external third of the eyebrow [1].

These cysts tend to present in the second or the third decades of life. The presentation depends on the site of the lesion, the size of the lesion, and the presence or absence of complications. The lesions tend to increase gradually in size, causing symptoms like pain. Dermoid cysts of the oral cavity usually present with asymptomatic masses, difficulties during chewing, dysarthria, and repeated infections, and when the cyst reach a large size may cause difficulties in breathing and swallowing [3, 4].

The differential diagnoses of sublingual tumors are many and may include both malignant and benign lesions. Malignant lesions include adenoid cystic carcinoma, mucoepidermoid carcinoma, acinic cell carcinoma, carcinoma ex pleomorphic adenoma, polymorphous low-grade adenocarcinoma, primary squamous cell carcinoma, salivary duct carcinoma, clear cell carcinoma, basal cell carcinoma, mucinous adenocarcinoma, and papillary cystadenocarcinoma, while benign lesions may include pleomorphic adenoma and basal cell adenoma [5].

The diagnosis is usually done by imaging because of the variety of differential diagnoses. Needle aspiration may be done, which is safe and effective in the diagnoses. CT-scan and MRI of the lesion are very helpful in localizing the exact site and size of the lesion, and also, imaging may help the surgeons in selecting the approach of the operation. Aspiration of the cyst contents. The recommended treatment of dermoid cysts is surgical excision, and lesions within the oral cavity are excised either with intraoral or extraoral approach. The prognosis is excellent after complete surgical excision, and the recurrence rate is very low [4, 6].

## 2. Case Presentation

A 13-year-old boy presented to the surgical clinic with a gradually enlarging sublingual mass over a period of 4 years. The mass was painless at start but in the last 3 months, it was associated with pain and difficulties during eating, dysarthria, and dysphagia.

**Figure 1:**
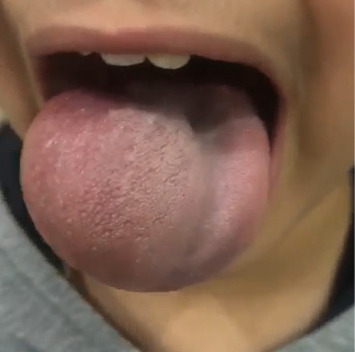
The swelling of the right side of the tongue.

**Figure 2:**
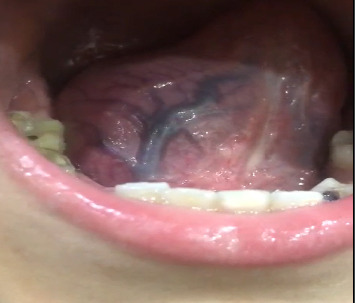
The swelling at the sublingual region.

The general examination was unremarkable and examination of the oral cavity showed a 5∗6 cm mass in the right side of the tongue and within the tongue. The mass was soft and nontender, fluctuation was positive, there was no pulsation over the mass, transillumination was negative, and other parts of the oral cavity were normal (Figures 1 and 2).

Neck examination was also normal with no enlargement of the cervical lymph nodes.

**Figure 3:**
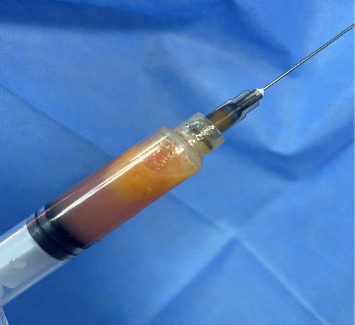
Aspiration of the cystic lesion using a syringe showing the typical yellow-straw–colored fluid containing cholesterol crystals.

Needle aspiration of the lesion was done with a syringe, which showed turbid yellow fluid (keratin-like substance) with no blood (Figure 3).

**Figure 4:**
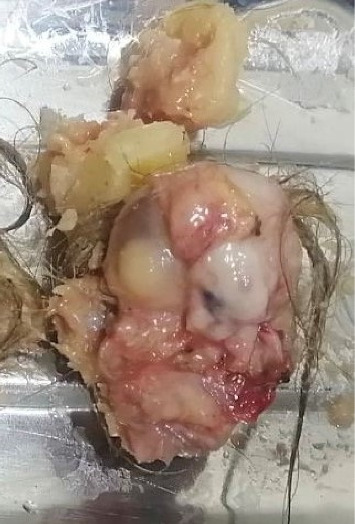
The contents of the cyst such as hair, sebum, and other different types of tissues.

The patient was prepared for surgery; during surgery, complete excision of the cyst was done, which was dermoid cyst of the tongue containing hair and fat. The patient was discharged on the same day of surgery with no postoperative events. Oral antibiotics and analgesics were prescribed (Figure 4).

There were no postoperative complications, and the sensory and motor examinations of the tongue were normal after surgery.

## 3. Discussion

Epidermoid cysts are benign lesions that may arise in different parts of the body and may be lined by simple squamous epithelium (epidermoid cyst), contain skin adnexa (“true” dermoid cyst), or may contain tissues of all the three germ layers and in such case, they are called teratoid cysts [7, 8].

Epidermoid cysts of the tongue are very rare and it may be congenital or acquired. The congenital ones are caused by failure of the separation of surface ectoderm or invagination of the surface ectoderm along the embryologic fusion lines. The acquired ones are caused by trauma, resulting in implantation of the epithelial elements into the deeper tissues and eventual growth of these tissues and gradual increase in size [9].

Needle aspiration may be done, which is safe and effective in the diagnosis, but may carry the risk of infection of the cyst contents, which may lead to abscess formation. Needle aspiration may show the typical yellow-straw–colored fluid containing cholesterol crystals, and examination of the contents under microscope may show anucleated squamous against an amorphous background [6].

Dermoid cysts of the oral cavity are principally divided into 3 types depending on the location, sublingual, submental, and submandibular. The most favored site of the oral cavity is the midline of the floor of the mouth, but cysts may also occur rarely in the buccal mucosa, the tongue, the lips, the uvula, and the temporo-mandibular joint. Patients with dermoid cysts of the oral cavity usually present with dysphagia, dysphonia, and dyspnea due to upward displacement the tongue. Growth in the inferior direction may give rise to the characteristic “double chin appearance” [2, 6].

The cysts are soft with doughy consistency, and they are well capsulated. A sudden increase in size may occur at the time of puberty because of the increase in the activity of the sebaceous glands and increased sebum production. The differential diagnoses of the dermoid cysts of the oral cavity include lympho-epithelial cyst, thyroglossal cyst, ranula, cystic hygroma, lymphangioma, and abscesses [2].

The histological examination of the cysts shows that the cyst often contain keratin, sebaceous glands, hair, nails, and fat globules and even cartilages may be present [2].

Recurrence of the cysts is very rare after complete surgical excision. The rate of malignant transformation of oral dermoid cysts is around 5%, and cases of teratoid type have been reported in literature [2].

Early diagnosis and precise excision of the cyst will result in excellent functional and esthetic outcomes. The majority of cysts can be excised through the oral cavity although cervical approach may be adopted for giant cysts [10, 11].

## Data Availability

The data used to support the findings of this study are available from the corresponding author upon reasonable request.
